# Gonadotropin receptor variants are linked to cumulative live birth rate after in vitro fertilization

**DOI:** 10.1007/s10815-018-1318-y

**Published:** 2018-09-19

**Authors:** I. Lindgren, H. Nenonen, E. Henic, L. Bungum, A. Prahl, M. Bungum, I. Leijonhufvud, I. Huhtaniemi, C. Yding Andersen, Y. Lundberg Giwercman

**Affiliations:** 1 Department of Translational Medicine, Molecular Genetic Reproductive Medicine, Clinical Research Centre, Lund University, Jan Waldenströms gata 35, Building 91, Plan 10, SE 21428 Malmö, Sweden; 2 Reproductive Medicine Centre, Skåne University Hospital, JanWaldenströms gata 47, Plan 3, SE 21428 Malmö, Sweden; 3 Department of Obstetrics and Gynecology, Herlev Hospital, Herlev Ringvej 75, 2730 Herlev, Denmark; 4 Hammersmith Campus, Institute of Reproductive and Developmental Biology, Imperial College London, London, SW7 2AZ UK; 5 Laboratory of Reproductive Biology, Rigshospitalet, Blegdamsvej 9, 2100 Copenhagen, Denmark

**Keywords:** FSH receptor, LHCG receptor, In vitro fertilization, Polymorphism, Infertility

## Abstract

**Purpose:**

The objective was to investigate if the gonadotropin receptor variants N680S (N: asparagine, S: serine, rs6166) in the follicle-stimulating hormone receptor (*FSHR*) and N312S (rs2293275) in the luteinizing hormone/human chorionic gonadotropin receptor (*LHCGR*) predicted cumulative live birth rate after in vitro fertilization (IVF).

**Methods:**

A total of 665 women were consecutively enrolled for IVF during the period 2007–2016. Inclusion criteria were < 40 years of age, body mass index < 30 kg/m^2^, non-smoking, regular menstruation cycle of 21–35 days, and bilateral ovaries. A blood sample was drawn for endocrine hormonal analysis and for DNA extraction with subsequent genotyping of the *FSHR* N680S and *LHCGR* N312S polymorphisms. Statistical analyses were done on all completed IVF cycles.

**Results:**

Women homozygous for S in both receptors combined (4S) had significantly higher live birth rate compared to those with other receptor variants when combining the first three IVF cycles (OR = 2.00, 95% CI [1.02, 3.92], *p* = 0.043). Cumulatively higher chance of live birth rate, during all IVF cycles, was also evident (HR = 1.89, 95% CI [1.00, 3.57], *p* = 0.049).

**Conclusions:**

Gonadotropin receptor variants are promising candidates for the prediction of the possibility to have a baby to take home after IVF treatment.

## Introduction

The gonadotropins follicle-stimulating hormone (FSH) and luteinizing hormone (LH) are both regulated by gonadotropin-releasing hormone (GnRH) and exert their actions via their specific membrane-bound receptors. The FSH receptor (FSHR) is expressed on ovarian granulosa cells and the LH/human chorionic gonadotropin (hCG) receptor (LHCGR) on granulosa, theca, and luteal cells [1]. Both receptors belong to the G protein-coupled receptor family and signal through the classical Gα_s_/3′,5′-cyclic adenosine monophosphate (cAMP)/protein kinase A pathway [2–4], but also through other signaling pathways as for example the adaptor protein, phosphotyrosine interaction, PH domain, and leucine zipper containing 1 (APPLI1)/inositol 1,4,5-triphosphate (IP_3_) signaling pathway [5]. In response to FSH, follicle recruitment takes place, whereas LH promotes the production of androgens, ovulation, and the maintenance of corpus luteum [6].

The *FSHR* gene is located on chromosome 2 and contains 10 exons. Exon 10 includes one common single nucleotide polymorphism (SNP) in amino acid position 680 (N680S, N: asparagine, S: serine; rs6166), and in Caucasian populations, approximately 20% are homozygous for S, whereas 30% are homozygous for N and 50% are heterozygous (N680S) [7, 8]. It has previously been shown that normally menstruating women have longer menstruation cycles if homozygous for S in *FSHR* N680S, which indicates a higher sensitivity threshold to FSH compared to women with the *FSHR* N680 genotype [9]. Higher basal FSH concentrations and lower estradiol production has in several clinical studies been associated with the *FSHR* S variant [9–13], and in previous studies on women undergoing in vitro *fertilization* (IVF), the *FSHR* N680S polymorphism has been described as a biomarker for decreased hormone sensitivity in controlled ovarian hyperstimulation (COH) prior to IVF, where decreased hormone sensitivity was found for women homozygous for S compared to women with other genotypes [10, 14, 15]. However, conflicting results have been produced [16, 17], and therefore, attempts have been made to further characterize the group of women that possibly could benefit from different stimulation regimens in COH.

The *LHCGR* gene, consisting of 11 exons, is located close to the *FSHR* gene on chromosome 2. One of the most studied polymorphic sites of the *LHCGR* gene is the N312S variant (rs2293275) in exon 10, also in this case changing N to S. In Caucasian populations, approximately 18% are homozygous for N312, 49% are heterozygous (N312S), and 33% are homozygous for S312 [18]. The *LHCGR* N312S variant has not been explored to the same extent as the *FSHR* in the context of COH prior to IVF. However, it was recently shown that women homozygous for S in both the abovementioned gonadotropin receptor polymorphisms had a fourfold increased chance of achieving pregnancy after the first IVF cycle, compared to women with other genotypes [19]. The objective of the current study was therefore to investigate if genotype, besides the impact on clinical parameters in connection with IVF, also was linked to live birth rate when taking all IVF cycles ever carried out into account.

## Materials and methods

### Subjects

During the period 2010–2016, *n* = 455 consecutively enrolled non-smoking women undergoing IVF were included in the study. The women were younger than 40 years of age at inclusion and had regular menstruation cycles of 21–35 days and bilateral ovaries. Body mass index (BMI) was < 30 kg/m^2^. The duration of infertility was at least 12 months, and couples underwent IVF with or without intracytoplasmic sperm injection (ICSI) per individually set guidelines. The cause of infertility could be any, providing that the women matched the abovementioned criteria. A venous blood sample was drawn for DNA extraction and subsequent genotyping of the *FSHR* and *LHCGR* polymorphisms.

An additional, independent cohort of 210 unselected women was included as a validation population, under the same conditions, during the period 2007–2015. The total study population thus comprised *n* = 665 women, and all women were of a Caucasian origin. The mean age of the women was 32.5 years (SD 3.8) on the day of inclusion (Table 1). Written informed consent was obtained from all participants. The study was approved by the regional ethical committee board in Lund, Sweden.

**Table 1:** Patient characteristics IVF cycle 1

		Age, mean (SD)	Follicle count, mean (SD)^a^	Retrieved oocytes, mean (SD)^b^	MII oocytes, mean (SD)^c^	GQE, mean (SD)^d^	Baseline AMH^e^, mean (SD)^f^	Baseline E2^g^, mean (SD)^h^	Baseline FSH^i^, mean (SD)^j^	Baseline LH^k^, mean (SD)^l^
*FSHR*	NN	33.1 (3.8)	12.6 (7.2)	11.0 (6.7)	8.2 (4.5)	2.4 (2.1)	31.2 (25.2)	432.1 (342.0)	6.5 (2.4)	10.2 (11.9)
	NS	32.2 (3.8)	12.1 (6.3)	10.7 (6.1)	7.1 (4.1)	2.1 (1.6)	29.5 (20.3)	406.5 (297.9)	6.3 (2.7)	8.6 (9.4)
	SS	32.6 (3.7)	11.1 (5.8)	10.0 (5.6)	6.1 (3.0)	2.1 (1.6)	31.5 (24.4)	431.1 (404.6)	6.4 (2.9)	10.4 (11.5)
	All	32.5 (3.8)	12.1 (6.5)	10.7 (6.2)	7.3 (4.1)	2.2 (1.8)	30.3 (22.4)	417.5 (329.4)	6.3 (2.7)	9.3 (10.5)
*p*		0.036	0.168	0.373	0.012	0.264	0.711	0.720	0.856	0.231
p*		–	0.149	0.347	0.009	0.216	0.511	0.813	0.878	0.232
*p* trend		–	–	–	0.003	–	–	–	–	–
*p* trend*		–	–	–	0.002	–	–	–	–	–
*LHCGR*	NN	32.3 (3.7)	12.3 (6.4)	11.1 (6.0)	6.9 (3.5)	1.9 (1.3)	32.6 (23.8)	419.8 (325.6)	6.3 (2.3)	8.6 (9.6)
	NS	32.4 (3.9)	11.7 (6.3)	10.3 (6.2)	7.1 (4.1)	2.1 (1.8)	29.5 (20.6)	395.0 (306.2)	6.5 (2.5)	8.8 (9.3)
	SS	32.7 (3.8)	12.5 (6.9)	10.9 (6.5)	7.7 (4.4)	2.4 (2.0)	30.1 (23.8)	445.5 (358.6)	6.1 (3.0)	10.4 (12.2)
	All	32.5 (3.8)	12.1 (6.5)	10.7 (6.2)	7.3 (4.1)	2.2 (1.8)	30.3 (22.4)	417.5 (329.4)	6.3 (2.7)	9.3 (10.5)
*p*		0.435	0.384	0.439	0.362	0.050	0.599	0.336	0.411	0.287
p*		–	0.349	0.419	0.313	0.043	0.590	0.437	0.382	0.286
*p* trend		–	–	–	–	0.015	–	–	–	–
*P* trend*		–	–	–	–	0.013	–	–	–	–
*FSHR/LHCGR*	0 S	32.9 (4.1)	13.0 (7.1)	10.8 (5.4)	8.5 (3.6)	1.8 (1.0)	33.4 (23.9)	399.7 (331.7)	6.0 (2.6)	6.1 (3.0)
	1 S	32.5 (3.8)	12.1 (6.5)	11.0 (6.4)	7.4 (4.3)	2.0 (1.7)	29.5 (20.3)	445.4 (362.4)	6.5 (2.4)	9.7 (11.3)
	2 S	32.5 (3.9)	12.2 (6.7)	10.7 (6.6)	7.1 (4.2)	2.4 (2.0)	31.7 (24.9)	391.5 (277.2)	6.4 (2.1)	9.3 (10.5)
	3 S	32.5 (3.8)	11.8 (6.3)	10.4 (5.9)	7.1 (4.1)	2.1 (1.6)	27.8 (19.4)	411.0 (305.2)	6.4 (3.6)	9.6 (9.9)
	4 S	32.7 (3.8)	12.0 (6.3)	10.3 (5.6)	7.1 (3.5)	2.3 (1.7)	33.3 (24.7)	530.3 (546.8)	5.5 (1.7)	9.8 (14.0)
	All	32.5 (3.8)	12.1 (6.5)	10.7 (6.2)	7.3 (4.1)	2.2 (1.8)	30.3 (22.4)	417.5 (329.4)	6.3 (2.7)	9.3 (10.5)
*p*		0.985	0.900	0.931	0.769	0.288	0.528	0.268	0.662	0.626
p*		–	0.889	0.931	0.760	0.287	0.594	0.268	0.664	0.627

^a^Data regarding follicle count was missing for 6 women. ^b^Data regarding retrieved oocytes was missing for 6 women. ^c^Data regarding MII oocytes was only present for those who underwent intracytoplasmic sperm injection (*n* = 317) and was hence missing for 348 women. ^d^Data regarding GQE was missing for 101 women. ^e^pmol/L. ^f^Data regarding baseline AMH was missing for 241 women. ^g^pmol/L. ^h^Data regarding baseline E2 was missing for 210 women. ^i^IU/L. ^j^Data regarding baseline FSH was missing for 322 women. ^k^IU/L. ^l^Data regarding baseline LH was missing for 211 women. *Adjusted for age. *AMH* Anti-Müllerian hormone, *E2* estradiol, *FSH* follicle-stimulating hormone, *FSHR* FSH receptor, *GQE* good quality embryos, *IVF* in vitro fertilization, *LH* luteinizing hormone, *LHCGR* LH/human chorionic gonadotropin receptor, *MII* metaphase II, *N* asparagine, *S* serine, *SD* standard deviation

### Patient treatment

#### First cycle

In the merged study cohort, 61% of the 665 women followed a standard long protocol, in which the GnRH agonist nafarelin (Synarela, Pfizer AB, Sollentuna, Sweden) was used in 40% of the women and the GnRH agonist buserelin (Suprefact, Sanofi AB, Stockholm, Sweden, and Suprecur, Sanofi AB) in the remaining 19 and 2%, respectively. The rest of the women followed a short protocol, in which the GnRH antagonist ganirelix (Orgalutran, Organon [Ireland] Ltd., Dublin, Ireland) was used. Two of the 665 women did not use a GnRH agonist or antagonist in the first IVF cycle, and data regarding GnRH agonist or antagonist was missing for one woman. The agonist treatment was initiated at day 21 (in a menstruation cycle of normal length) in the cycle directly prior to the cycle with hormonal stimulation, and the antagonist treatment was initiated at day 2 in the same cycle as the hormonal stimulation. During the first IVF cycle, ovarian stimulation was induced by individually set doses of stimulation products, presented in Table 2, using either follitropin alpha (GONAL-f, Merck-Serono, Darmstadt, Germany), follitropin beta (Puregon, Organon [Ireland] Ltd.), urofollitropin (Fostimon, Institute Biochimique SA [IBSA] Farmaceutici Italia Srl, Lodi, Italy), menotropin (Menopur, Ferring Läkemedel AB, Malmö, Sweden), corifollitropin alpha (Elonva, Merck Sharp & Dohme [MSD] Sweden AB, Stockholm, Sweden), or estradiol (Progynon, Bayer AB, Solna, Sweden). The development of the ovarian follicles was monitored by vaginal ultrasound on days 6–8 of ovarian stimulation, and if needed, the hormonal dose was adjusted to generate at least three follicles. Independent of long or short stimulation protocol, when three or more mature follicles (≥ 18 mm) were confirmed by vaginal ultrasound, hCG was administered and oocyte retrieval was performed approximately 35 h later. Embryos were scored according to the guidelines by Gardner and Schoolcraft [20–22]. Pregnancy was confirmed by an hCG test 14 days after embryo transfer.

**Table 2:** Stimulation protocols

	First IVF cycle			Second IVF cycle			Third IVF cycle		
	First cohort	Validation cohort	Merged cohort^a^	First cohort	Validation cohort	Merged cohort^b^	First cohort	Validation cohort	Merged cohort^c^
All treatments, *n* (%)	455 (100)	204 (97)	659 (99)	263 (100)	158 (100)	421 (99)	149 (100)	111 (100)	260 (100)
Mean total dose (SD)	1655 (753)	2158 (895)	1815 (829)	1989 (1052)	2498 (1063)	2181 (1083)	2212 (1217)	2496 (1068)	2335 (1163)
Follitropin alpha, *n* (%)	322 (71)	173 (82)	497 (75)	154 (59)	119 (74)	273 (64)	81 (54)	79 (71)	160 (61)
Mean total dose (SD), IU	1639 (698)	2116 (890)	1805 (797)	1825 (904)	2472 (1093)	2116 (1037)	1919 (1077)	2397 (981)	2157 (1058)
Follitropin beta, *n* (%)	96 (21)	23 (11)	117 (18)	37 (14)	14 (9)	51 (12)	12 (8)	10 (9)	22 (8)
Mean total dose (SD), IU	1653 (734)	2002 (757)	1733 (748)	1853 (949)	2107 (811)	1943 (913)	2052 (794)	1817 (670)	1945 (733)
Urofollitropin, *n* (%)	20 (4)	–	20 (3)	6 (2)	2 (1)	8 (2)	7 (5)	1 (1)	8 (3)
Mean total dose (SD), IU	1774 (791)	–	1774 (791)	2446 (1439)	1275 (106)	2153 (1332)	4264 (1348)	4500 (0)	4294 (1251)
Menotropin, *n* (%)	11 (2)	8 (4)	19 (3)	64 (24)	21 (13)	85 (20)	47 (32)	20 (18)	67 (26)
Mean total dose (SD), IU	2726 (1049)	2981 (759)	2834 (923)	2478 (1219)	3008 (903)	2612 (1172)	2546 (1116)	3244 (1020)	2754 (1127)
Corifollitropin alpha, *n* (%)	5 (1)	–	5 (1)	–	–	–	–	–	–
Mean total dose (SD), μg	150 (0)	–	150 (0)	–	–	–	–	–	–
Estradiol, *n* (%)	1 (0)	–	1 (0)	2 (1)	2 (1)	4 (1)	2 (1)	1 (1)	3 (1)
Mean total dose (SD), pmol/L	84 (0)	–	84 (0)	114 (17)	852 (1129)	483 (779)	48 (59)	102 (0)	66 (52)

^a^Data regarding stimulation product was missing for six women in the first IVF cycle. ^b^Data regarding total dose of stimulation product was missing for one woman and data regarding stimulation product was missing for four women in the second IVF cycle. ^c^Data regarding the total dose of stimulation product was missing for one woman in the third cycle. *IVF* in vitro fertilization, *SD* standard deviation

#### Cycles 2–7

**Fig. 1:**
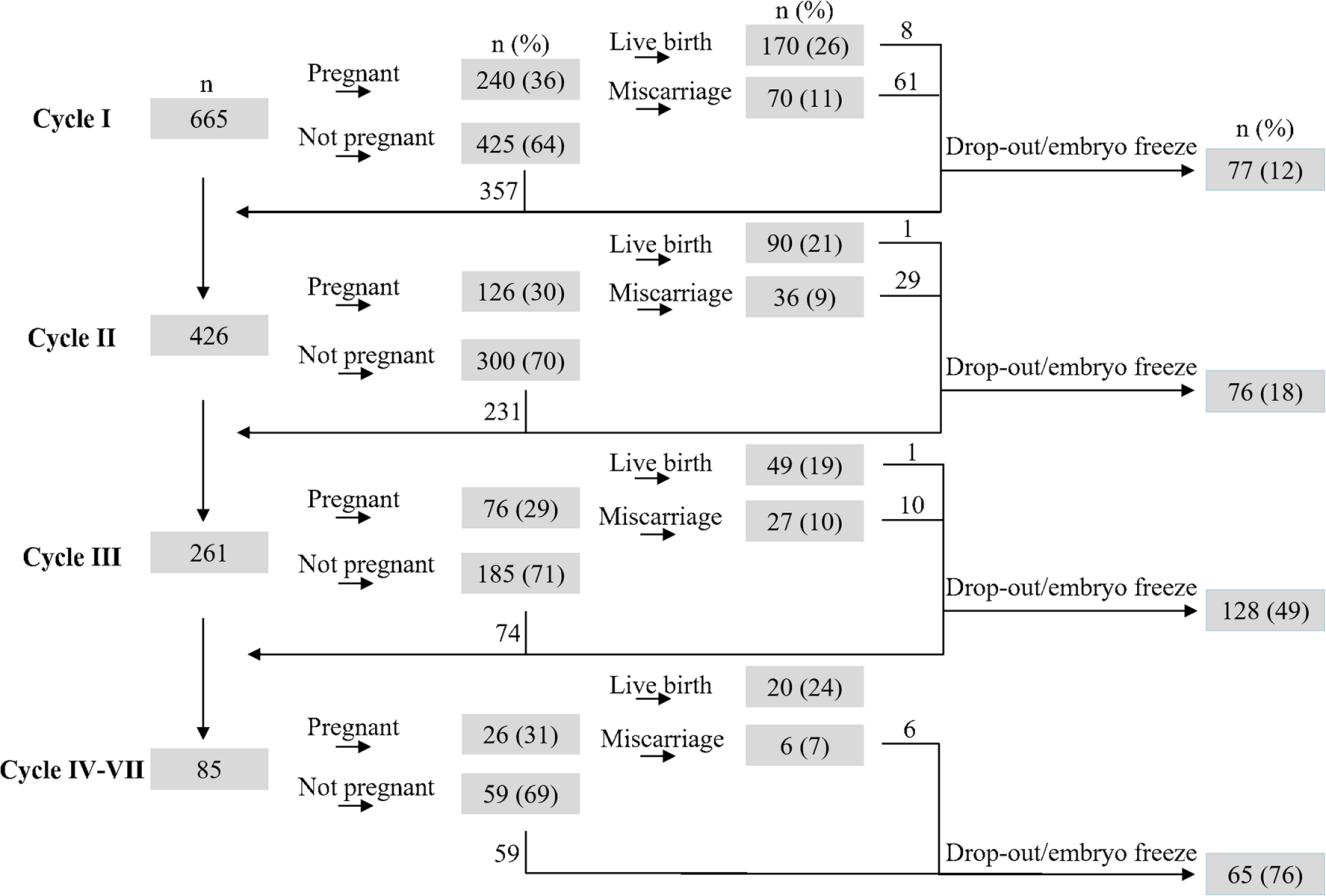
Flow chart of women participating in the study. Gray boxes indicate number and frequency (if stated) of women at each event. Numbers alongside the arrows indicate the number of women continuing to the next cycle from the event where the arrow starts

Of the *n* = 665 women in the merged cohort, *n* = 426 women (64%) completed a second IVF cycle, and *n* = 261 (39%) continued to a third (Fig. 1). Of these, *n* = 85 (13%) sustained 4–7 cycles. Hormonal treatments are stated in Table 2.

### Genotyping of *FSHR* N680S and *LHCGR* N312S

Genomic DNA was extracted from peripheral leukocytes using standard procedures. The polymorphism in amino acid position 680 of the *FSHR* gene was genotyped by allele-specific PCR using two amplification reactions for each subject, each containing one wild-type or one mutant-specific primer at concentrations of 0.12 μM as well as two flanking primers (forward and reverse, respectively) at concentrations of 0.3 μM (wild-type specific primer: 5′-GACAAGTATGTAAGTGGAACCAT-3′, mutant-specific primer: 5′-GACAAGTATGTAAGTGGAACCAC-3′, forward flanking primer: 5′-TTCACCCCATCAACTCCTGT-3′, reverse flanking primer: 5′-TCCTGGCTCTGCCTCTTACA-3′; Invitrogen, Stockholm, Sweden). Amplifications were performed in a total volume of 50 μL, containing, in addition to the primers, 10 mM Tris-HCl (Saveen & Werner AB, Limhamn, Sweden) pH 9.1, 45 mM KCl (ICN Biomedicals INC., Aurora, OH, USA), 0.1% *w*/*v* Tween 20 (Scharlau Chemie S.A., Barcelona, Spain), 1.5 mM MgCl_2_ (Sigma-Aldrich Sweden AB, Stockholm, Sweden), 200 μM of each dNTP (dATP, dCTP, dGTP, and dTTP, Fermentas, Sankt Leon-Rot, Germany), 1 U Dynazyme™ II DNA polymerase (Thermo Fisher Scientific Inc., Waltham, MA, USA), and 200 ng template DNA. Amplifications were carried out for 26 cycles, each including denaturation for 1 min at 96 °C, primer annealing at 56 °C for 30 s and extension for 3 min at 72 °C. An initial hot start at 96 °C and a final extension for 5 min at 72 °C were also used. The results from the allele-specific PCR were confirmed by direct sequencing of three purified PCR samples of each genotype on an eight-capillary Applied Biosystems sequencing gear (Applied Biosystems, Stockholm, Sweden). The PCR samples were purified using a DNA purification kit (ExtractMe DNA clean-up kit, Biolab Innovative Research Technologies [Blirt] S.A., DNA-Gdańsk, Gdańsk, Poland). The polymorphism in amino acid position 312 of the *LHCGR* was analyzed by PCR and direct sequencing of the PCR product. The PCR reactions were performed in a total volume of 50 μL containing 0.4 μM of the forward primer 5′-TGTTGACCATGTGACTAGGGA and 0.4 μM of the reverse primer 5′-ACTCTCTCCTCAGGAAGCAT (Invitrogen), 10 mM Tris-HCl (Saveen & Werner AB) pH 9.1, 45 mM KCl (ICN Biomedicals INC.), 0.01% *w*/*v* Tween 20 (Scharlau Chemie S.A.), 1.5 mM MgCl_2_ (Sigma-Aldrich Sweden AB), 200 μM of each dNTP (Fermentas), 1 U Dynazyme™ II DNA polymerase (Thermo Fisher Scientific Inc.), and 200 ng template DNA. The amplification program was initiated by a denaturation step at 96 °C for 10 min, followed by 37 amplification cycles, each consisting of denaturation at 96 °C for 1 min, annealing at 61 °C for 30 s and elongation at 72 °C for 3 min. A final elongation at 72 °C for 7 min was applied. The PCR product was purified using a DNA purification kit (ExtractMe DNA clean-up kit, Blirt S.A., DNA-Gdańsk) and directly sequenced on an eight-capillary Applied Biosystems sequencing gear (Applied Biosystems).

### Statistical analysis

Allele frequencies of the two polymorphisms were analyzed in comparison to the control populations using a chi-squared test. The *LHCGR* N312S polymorphism was analyzed against a normal population of 3794 Caucasians [23], while the *FSHR* polymorphism was tested against a normal population of 1431 Caucasians [8]. When analyzing the differences in clinical parameters between genotypes, all groups were tested separately, i.e., homozygous N versus heterozygous versus homozygous S. Since heterodimerization between the receptors may occur [24, 25], a combination of the genotypes of both receptors was also used, i.e., NN/NN versus NS/NS versus SS/SS. Comparisons among clinical parameters and hormonal doses and agents given prior to IVF were carried out using a univariate analysis of variance and a chi-squared test. Comparisons of incidence of live births and genotype were done using a logistic regression analysis. The incidence of live births in relation to increasing number of S was calculated using a linear regression analysis, as was number of metaphase II (MII) oocytes in relation to *FSHR* genotype and number of good quality embryos in relation to *LHCGR* genotype. When analyzing the chance of live birth rate in seven IVF cycles, a Cox regression analysis was used. Age (as a continuous variable) was considered as a confounding factor when analyzing the differences in clinical parameters between the groups.

Since the study was performed on candidate genes, no correction for mass significance was done [26].

Data were analyzed using SPSS software version 22, 24, and 25 (SPSS, Inc., Chicago, IL, USA). A *p* value < 0.05 was considered statistically significant.

## Results

### Patient characteristics

There was no difference in age between genotype groups except for a marginal difference in age linked to the *FSHR* variants in IVF cycle 1 (Table 1). Follicle counts did not differ, neither regarding each receptor per se nor combined. A difference in number of MII oocytes was found among women with different variants of the *FSHR* N680S in cycle I (NN, 8.2 ± 4.5; NS, 7.1 ± 4.1; SS, 6.1 ± 3.0; unadjusted, *p* = 0.012; adjusted, *p* = 0.009), and there was also a trend with decreasing number of MII oocytes with decreasing number of S (unadjusted: *B* = − 1.07, 95% CI for *B* [− 1.77, − 0.365], *p* trend = 0.003; adjusted: *B* = − 1.11, 95% CI for *B* [− 1.81, − 0.401], *p* trend = 0.002). Data on MII oocytes was only present for those that underwent ICSI (*n* = 317) and was hence missing for 348 women. There was also a difference in good quality embryos retrieved from women after hormonal ovarian stimulation was evident regarding the *LHCGR* per se (NN, 1.9 ± 1.3; NS, 2.1 ± 1.8; SS, 2.4 ± 2.0; unadjusted, *p* = 0.050; adjusted, *p* = 0.043), and a trend with increasing number of good quality embryos and increasing number of S (unadjusted: *B* = 0.257, 95% CI for *B* [0.050, 0.469], *p* trend = 0.015; adjusted: *B* = 0.264, 95% CI for *B* [0.056, 0.471], *p* trend = 0.013) was also evident. The follicle, oocyte, and embryo counts did not differ with respect to the use of recombinant or urine-derived stimulation product (*p* = 0.886 for number of follicles; *p* = 0.243 for number of retrieved oocytes; *p* = 0.581 for number of MII oocytes; *p* = 0.251 for number of good quality embryos). No other differences regarding background characteristics were found between women from different genotype groups in the first IVF cycle, and in subsequent cycles, no differences were found regarding background characteristics among women from different genotype groups, except for a minimal difference in age linked with *FSHR* variant in cycle 2 (NN, 34.0 ± 3.7; NS, 32.9 ± 3.9; SS, 33.6 ± 3.3; *p* = 0.041). Eighty-seven percent of the embryos were transferred back to the woman on day 3 in IVF cycle 1, and 13% were transferred at day 5. In cycle 2, 86% of the embryos were transferred at day 3 and 16% were transferred at day 5, and in cycle, 3 83% of the embryos were transferred at day 3 and 17% were transferred at day 5.

### Genotyping

Allele frequencies and genotype distributions were of expected proportions (Table 3), similar to previously reported study populations (*p* = 0.776 for *FSHR* and *p* = 0.666 for *LHCGR*) [8, 23]. The allele frequencies of both polymorphisms were in Hardy-Weinberg equilibrium, *χ*^2^ = 0.770, *p* > 0.05, for *FSHR* N680S and *χ*^2^ = 0.342, *p* > 0.05, for *LHCGR* N312S.

**Table 3:** Allele frequencies and genotype distributions for *FSHR* and *LHCGR*

		Allele frequencies, (%)		Genotype distribution, *n* (%)		
		A	G	N/N	N/S	S/S
*FSHR* N680S	First cohort	55	45	124 (27)	257 (57)	74 (16)
	Validation cohort	58	42	73 (35)	99 (47)	38 (18)
	Merged cohort	56	44	197 (30)	356 (54)	112 (17)
*LHCGR* N312S	First cohort	40	60	79 (17)	209 (46)	167 (37)
	Validation cohort	37	63	32 (15)	90 (43)	88 (42)
	Merged cohort	39	61	111 (17)	299 (45)	255 (38)

*A* adenine, *FSHR* follicle-stimulating hormone receptor, *G* guanine, *LHCGR* luteinizing hormone/human chorionic gonadotropin receptor, *N* asparagine, *S* serine

### Hormonal treatment

For the *FSHR* N680S and the *LHCGR* N312S variants, no differences between carriers of different genotypes in terms of total hormone dose were evident in the first or second IVF cycle (Table 4). In the third IVF cycle, the group of women that were homozygous for N in both receptors was treated with a lower total dose of hormone for ovarian stimulation (*p* = 0.003). There were, however, no differences between genotypes in relation to the use of hormonal agents (Table 5).

**Table 4:** Total hormone doses according to genotype

		Total dose first cycle, mean (SD)	*p*	p*	p*^#^	Total dose second cycle, mean (SD)^a^	*p*	p*	p*^#^	Total dose third cycle, mean (SD)^b^	*p*	p*	p*^#^
*FSHR* N680S	NN	1846 (752)	0.166	0.327	–	2211 (996)	0.928	0.711		2256 (1183)	0.536	0.261	–
	NS	1763 (808)				2174 (1156)				2412 (1206)			
	SS	1924 (1001)				2155 (1010)				2238 (944)			
	All	1815 (829)				2181 (1083)				2335 (1163)			
*LHCGR* N312S	NN	1820 (955)	0.885	0.520	–	1999 (1036)	0.264	0.345		2022 (1119)	0.079	0.138	–
	NS	1830 (759)				2218 (1034)				2443 (1116)			
	SS	1795 (852)				2228 (1166)				2379 (1234)			
	All	1815 (829)				2181 (1083)				2335 (1163)			
*FSHR/LHCGR*	0 S	1724 (866)	0.666	0.539	0.339	1862 (1039)	0.625	0.460	0.082	1562 (975)	0.049	0.034	0.003
	1 S	1880 (828)				2197 (927)				2372 (1131)			
	2 S	1798 (795)				2227 (1143)				2437 (1182)			
	3 S	1783 (758)				2163 (1106)				2383 (1189)			
	4 S	1929 (1266)				2259 (1199)				2005 (787)			
	All	1815 (829)				2181 (1083)				2335 (1163)			

^a^Data regarding the total dose of stimulation product was missing for one woman in the second IVF cycle. ^b^Data regarding the total dose of stimulation product was missing for one woman in the third cycle. *Adjusted for age. ^#^0S versus all. *FSHR* follicle-stimulating hormone receptor, *LHCGR* luteinizing hormone/human chorionic gonadotropin receptor, *N* asparagine, *S* serine, *SD* standard deviation

**Table 5:** Hormonal agents according to *FSHR/LHCGR* genotype

		Follitropin alpha		Follitropin beta		Urofollitropin		Menotropin		Corifollitropin alpha		Estradiol		Total *n*	*p*
Cycle 1^a^		*n*	%	*n*	%	*n*	%	*n*	%	*n*	%	*n*	%		0.882
*FSHR/LHCGR*	0 S	23	66	9	26	1	3	2	6	–	–	–	–	35	
	1 S	110	80	24	17	2	1	2	1	–	–	–	–	138	
	2 S	199	77	42	17	8	3	7	3	2	1	1	–	259	
	3 S	137	73	33	18	9	5	6	3	3	2	–	–	188	
	4 S	28	72	9	77	–	–	2	5	–	–	–	–	39	
	All	497	75	117	18	20	3	19	3	5	1	1	–	659	
Cycle 2^b^		*n*	%	*n*	%	*n*	%	*n*	%	*n*	%	*n*	%		0.520
*FSHR/LHCGR*	0 S	18	72	4	16	–	–	3	12	–	–	–	–	25	
	1 S	67	73	8	9	2	2	15	16	–	–	–	–	92	
	2 S	104	64	22	14	2	1	31	19	–	–	3	2	162	
	3 S	76	61	14	11	4	3	29	23	–	–	1	1	124	
	4 S	9	47	3	16	–	–	7	37	–	–	–	–	19	
	All	274	65	51	12	8	2	85	20	–	–	4	1	422	
Cycle 3		*n*	%	*n*	%	*n*	%	*n*	%	*n*	%	*n*	%		0.173
*FSHR/LHCGR*	0 S	13	72	1	6	–	–	3	17	–	–	1	6	18	
	1 S	40	62	4	6	2	3	18	28	–	–	1	2	65	
	2 S	64	62	9	9	2	2	28	27	–	–	1	1	104	
	3 S	40	60	6	9	4	6	17	25	–	–	–	–	67	
	4 S	4	60	2	29	–	–	1	14	–	–	–	–	7	
	All	161	62	22	8	8	3	67	26	–	–	3	1	261	

^a^Data regarding stimulation product was missing for six women in the first IVF cycle. ^b^Data regarding stimulation product was missing for four women in IVF cycle two. *FSHR* follicle-stimulating hormone receptor, *LHCGR* luteinizing hormone/human chorionic gonadotropin receptor, *S* serine

### Pregnancy and live birth rate

In the present study, 240 of the 665 women (36%) achieved a pregnancy (defined by a positive hCG test) in the first IVF cycle, and 170 of these (71%) gave birth to a live-born baby (Fig. 1). In the second cycle, 126 of the 426 women (30%) achieved pregnancy and 90 women (71%) delivered a live-born baby. Seventy-six of the 261 women (29%) in the third cycle achieved pregnancy and 49 of those (64%) had a live-born baby. The cumulative pregnancy rate was 33% for all IVF cycles, and the cumulative live birth rate was 23%. In total, 70% of the women achieved a pregnancy after all IVF cycles, and 49% gave birth to a live-born baby. The live birth rates were not dependent on which protocol (agonist/antagonist) that was used (IVF cycle 1—unadjusted *p* = 0.227, adjusted *p* = 0.832; IVF cycle 2—unadjusted: *p* = 0.631, adjusted *p* = 0.645; IVF cycle 3—unadjusted *p* = 0.265, adjusted *p* = 0.461; IVF cycle 4—unadjusted *p* = 0.607, adjusted *p* = 0.639; IVF cycle 5—unadjusted *p* = 0.250, adjusted *p* = 0.399; IVF cycle 6—unadjusted *p* = 0.516, adjusted *p* = 0.837; IVF cycle 7—unadjusted *p* = 0.667, not adjusted because of too few subjects).

**Fig. 2:**
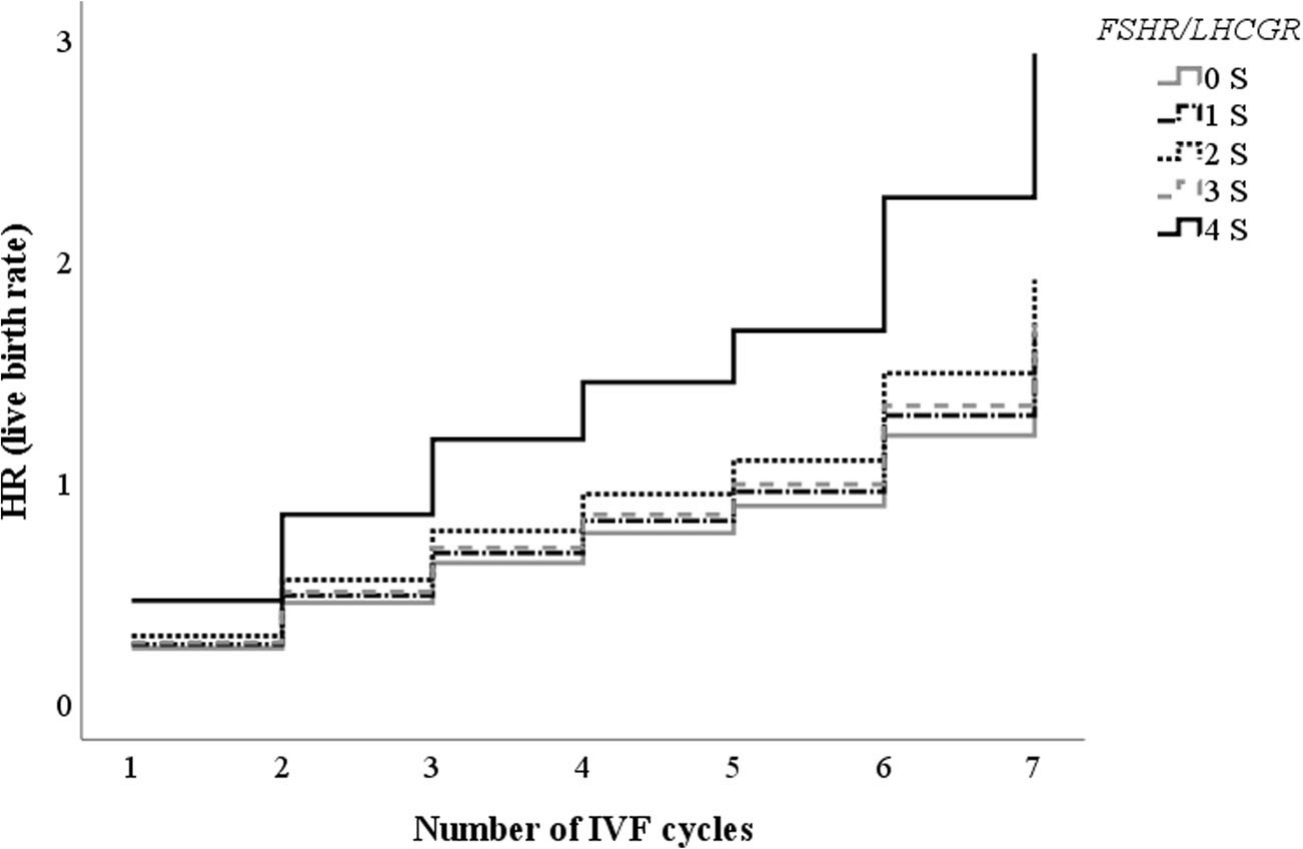
Cumulative hazard for live birth rate in relation to the number of S in *FSHR* N680S/*LHCGR* N312S. Adjusted for age. FSHR, follicle-stimulating hormone receptor; HR, hazard ratio; IVF, in vitro fertilization; LHCGR, LH/human chorionic gonadotropin receptor; S, serine

When analyzing live birth rate in IVF cycles 1–3, an association with number of S in both gonadotropin receptors combined and live birth rate was found; women homozygous for S had the highest chance to deliver a live-born baby (0 S 43%, 1 S 45%, 2 S 47%, 3 S 44%, 4 S 62%; unadjusted: OR = 1.94, 95% CI [0.998, 3.77], *p* = 0.051; adjusted: OR = 2.00, 95% CI [1.02, 3.92], *p* = 0.043). A higher chance of live birth rate for all IVF cycles was also evident for women homozygous for S in both receptors (unadjusted: HR = 1.83, 95% CI [0.970, 3.45], *p* = 0.062; adjusted: HR = 1.89, 95% CI [1.00, 3.57], *p* = 0.049; Fig. 2).

The *FSHR* per se was not associated with live birth rate, but in contrast, the *LHCGR* was. However, this was only noted in the first IVF cycle, in which a linear association with live birth rate with increasing number of S in the *LHCGR* N312S variant was evident (0 S 19%, 1 S 25%, 2 S 29%; unadjusted: OR = 0.045, 95% CI [− 0.001, 0.092], *p* trend = 0.057; adjusted: OR = 0.051, 95% CI [0.005, 0.097], *p* trend = 0.029).

## Discussion

The main finding of the present study was that in the first three IVF cycles, women homozygous for S in both *FSHR* N680S and *LHCGR* N312S had approximately 40% higher chance of having a baby compared to those with other genetic variants. In a cumulative analysis of all IVF cycles in this cohort, women with S in both receptors still had an advantage with respect to live birth rate, with overall almost doubled chance of giving birth to a live-born baby compared to women with N in the same positions.

The current results support the previous report on substantially higher pregnancy chance in homozygous S-carriers [19] and emphasize the importance of not only of the *FSHR*, but also of the *LHCGR* in IVF outcomes. The LHCGR component seems to require a higher hormone dose during stimulation, but in return, more good quality embryos after ovarian stimulation are produced by women with S in the *LHCGR* N312S position.

The finding that the women homozygous for S in both receptors became pregnant and delivered babies at a higher rate than women with other receptor variants is not easy to explain. It is, however, a well-documented phenomenon that G protein-coupled receptors can form dimers, both homo- and heterodimers, and even oligomers, which in some cases show different functional properties compared to the individual G protein-coupled receptors [27]. It seems plausible that also the FSHR and LHCGR can occur as structural or functional dimers enabling crosstalk upon signaling. As an example, it has previously been shown that increased density of the LHCGR resulted in a diminution of FSH-stimulated cAMP production in granulosa cells [28], and recently, also cross-reduction of signaling and heterodimerization between the human FSHR and LHCGR was demonstrated [24, 25]. Overall, neither the *FSHR* nor the *LHCGR* per se was linked to higher birth rate, except for the *LHCGR* which was associated with increased birth rate with increasing number of S in the first IVF cycle. However, the relationship was stronger when combining the receptors, which indicates the importance of both receptors for live birth rate.

In transgenic mice, co-expressing two functionally defective LHCGRs, one being binding deficient and the other one signaling deficient, receptor signaling by functional complementation was established [29]. It has also been demonstrated that FSHR/LHCGR heterodimers could work in the same fashion [25]. Whether the subtle functional alterations induced by *LHCGR* and *FSHR* polymorphisms could synergize as heterodimers is currently not known, but reasonable to hypothesize. In such case, the homozygous S312 variant, residing in the extracellular domain of the LHCGR, would imply intensification in hormone binding properties, whereas the FSHR S680 variant, located in the intracellular domain of the receptor, would ensure enhanced signaling in response to high doses of hormone.

Di- or oligomerization could also be the underlying mode of action for ligand promiscuity, as was shown regarding the FSHR, which was sensitized by hCG due to the variants in the serpentine part of the receptor [30]. The hormonal dose is low under physiological conditions and ensures specificity for distinct receptors but is high during the first trimester of normal human pregnancy as well as in IVF trials, and may therefore be the cause of promiscuous receptor binding. Whether this is applicable also to stimulation with FSH is currently not known. In the present study, almost all women were stimulated with recombinant FSH. We can therefore not draw firm conclusions regarding promiscuous binding, different agents, or combinations of such. Further research regarding the benefits of different therapy modalities in combination with the SNP profile is therefore warranted.

The strength of the study was the large cohort of consecutively enrolled patients. These women were hence not selected for the study, but an ordinary cohort of women visiting a fertility clinic. The findings can therefore be generally applied. Moreover, the method for genotyping was very accurate. A limitation was the lack of initial treatment doses and information on fresh and frozen-thawed embryos.

## Conclusions

In conclusion, in this large cohort of consecutively enrolled women, those homozygous for S in both *FSHR* N680S and *LHCGR* N312S had almost doubled chance of giving birth to a live-born baby after IVF compared to women homozygous for N. If the gonadotropin receptor polymorphisms were recognized as biomarkers in IVF trials, not only more effective treatment strategies could be developed in order to achieve pregnancies, but genotype could also be used in prediction of the chance to have a baby to take home.

## References

[1] Camp TA, Rahal JO, Mayo KE (1991). Cellular localization and hormonal regulation of follicle-stimulating hormone and luteinizing hormone receptor messenger RNAs in the rat ovary. Mol Endocrinol.

[2] Dattatreyamurty B, Figgs LW, Reichert LE (1987). Physical and functional association of follitropin receptors with cholera toxin-sensitive guanine nucleotide-binding protein. J Biol Chem.

[3] Dattatreyamurty B, Schneyer A, Reichert LE (1986). Solubilization of functional and stable follitropin receptors from light membranes of bovine calf testis. J Biol Chem.

[4] Means AR, MacDougall E, Soderling TR, Corbin JD (1974). Testicular adenosine 3′:5′-monophosphate-dependent protein kinase. Regulation by follicle-stimulating hormone. J Biol Chem.

[5] Thomas RM, Nechamen CA, Mazurkiewicz JE, Ulloa-Aguirre A, Dias JA (2011). The adapter protein APPL1 links FSH receptor to inositol 1,4,5-trisphosphate production and is implicated in intracellular Ca(2+) mobilization. Endocrinology.

[6] La Marca A, Sighinolfi G, Argento C, Grisendi V, Casarini L, Volpe A et al. Polymorphisms in gonadotropin and gonadotropin receptor genes as markers of ovarian reserve and response in in vitro fertilization. Fertil Steril 2013;99(4):970–978.e1. 10.1016/j.fertnstert.2013.01.086. 10.1016/j.fertnstert.2013.01.086 23380184

[7] Lindgren I, Giwercman A, Axelsson J, Lundberg Giwercman Y (2012). Association between follicle-stimulating hormone receptor polymorphisms and reproductive parameters in young men from the general population. Pharmacogenet Genomics.

[8] Kuijper EA, Blankenstein MA, Luttikhof LJ, Roek SJ, Overbeek A, Hompes PG (2010). Frequency distribution of polymorphisms in the FSH receptor gene in infertility patients of different ethnicity. Reprod BioMed Online.

[9] Greb RR, Grieshaber K, Gromoll J, Sonntag B, Nieschlag E, Kiesel L, Simoni M (2005). A common single nucleotide polymorphism in exon 10 of the human follicle stimulating hormone receptor is a major determinant of length and hormonal dynamics of the menstrual cycle. J Clin Endocrinol Metab.

[10] Sudo S, Kudo M, Wada S, Sato O, Hsueh AJ, Fujimoto S (2002). Genetic and functional analyses of polymorphisms in the human FSH receptor gene. Mol Hum Reprod.

[11] de Castro F, Moron FJ, Montoro L, Galan JJ, Hernandez DP, Padilla ES (2004). Human controlled ovarian hyperstimulation outcome is a polygenic trait. Pharmacogenetics.

[12] Behre HM, Greb RR, Mempel A, Sonntag B, Kiesel L, Kaltwasser P (2005). Significance of a common single nucleotide polymorphism in exon 10 of the follicle-stimulating hormone (FSH) receptor gene for the ovarian response to FSH: a pharmacogenetic approach to controlled ovarian hyperstimulation. Pharmacogenet Genomics.

[13] Lledo B, Guerrero J, Turienzo A, Ortiz JA, Morales R, Ten J (2013). Effect of follicle-stimulating hormone receptor N680S polymorphism on the efficacy of follicle-stimulating hormone stimulation on donor ovarian response. Pharmacogenet Genomics.

[14] Perez Mayorga M, Gromoll J, Behre HM, Gassner C, Nieschlag E, Simoni M (2000). Ovarian response to follicle-stimulating hormone (FSH) stimulation depends on the FSH receptor genotype. J Clin Endocrinol Metab.

[15] Yao Y, Ma CH, Tang HL, Hu YF (2011). Influence of follicle-stimulating hormone receptor (FSHR) Ser680Asn polymorphism on ovarian function and in-vitro fertilization outcome: a meta-analysis. Mol Genet Metab.

[16] Mohiyiddeen L, Newman WG, Cerra C, McBurney H, Mulugeta B, Roberts SA, Nardo LG (2013). A common Asn680Ser polymorphism in the follicle-stimulating hormone receptor gene is not associated with ovarian response to gonadotropin stimulation in patients undergoing in vitro fertilization. Fertil Steril.

[17] Falconer H, Andersson E, Aanesen A, Fried G (2005). Follicle-stimulating hormone receptor polymorphisms in a population of infertile women. Acta Obstet Gynecol Scand.

[18] Valkenburg O, Uitterlinden AG, Piersma D, Hofman A, Themmen AP, de Jong FH et al. Genetic polymorphisms of GnRH and gonadotrophic hormone receptors affect the phenotype of polycystic ovary syndrome. Hum Reprod (Oxford, England). 2009;24(8):2014–2022. 10.1093/humrep/dep113. 10.1093/humrep/dep113 19403562

[19] Lindgren I, Baath M, Uvebrant K, Dejmek A, Kjaer L, Henic E et al. Combined assessment of polymorphisms in the LHCGR and FSHR genes predict chance of pregnancy after in vitro fertilization. Hum Reprod (Oxford, England). 2016;31(3):672–683. 10.1093/humrep/dev342. 10.1093/humrep/dev342 26769719

[20] Gardner DK, Schoolcraft WB (1999). Culture and transfer of human blastocysts. Curr Opinion Obstet Gynecol.

[21] Gardner DK, Lane M, Stevens J, Schlenker T, Schoolcraft WB (2000). Blastocyst score affects implantation and pregnancy outcome: towards a single blastocyst transfer. Fertil Steril.

[22] Gardner DK, Lane M, Schoolcraft WB (2002). Physiology and culture of the human blastocyst. J Reprod Immunol.

[23] Piersma D, Verhoef-Post M, Look MP, Uitterlinden AG, Pols HA, Berns EM (2007). Polymorphic variations in exon 10 of the luteinizing hormone receptor: functional consequences and associations with breast cancer. Mol Cell Endocrinol.

[24] Feng X, Zhang M, Guan R, Segaloff DL (2013). Heterodimerization between the lutropin and follitropin receptors is associated with an attenuation of hormone-dependent signaling. Endocrinology.

[25] Jonas KC, Chen S, Virta M, Mora J, Franks S, Huhtaniemi I (2018). Temporal reprogramming of calcium signalling via crosstalk of gonadotrophin receptors that associate as functionally asymmetric heteromers. Sci Rep.

[26] Cordell HJ, Clayton DG (2005). Genetic association studies. Lancet (London, England).

[27] Rivero-Muller A, Jonas KC, Hanyaloglu AC, Huhtaniemi I (2013). Di/oligomerization of GPCRs-mechanisms and functional significance. Prog Mol Biol Transl Sci.

[28] Donadeu FX, Ascoli M (2005). The differential effects of the gonadotropin receptors on aromatase expression in primary cultures of immature rat granulosa cells are highly dependent on the density of receptors expressed and the activation of the inositol phosphate cascade. Endocrinology.

[29] Rivero-Muller A, Chou YY, Ji I, Lajic S, Hanyaloglu AC, Jonas K (2010). Rescue of defective G protein-coupled receptor function in vivo by intermolecular cooperation. Proc Natl Acad Sci of the United States of America.

[30] Costagliola S, Urizar E, Mendive F, Vassart G. Specificity and promiscuity of gonadotropin receptors. Reproduction (Cambridge, England). 2005;130(3):275–281. 10.1530/rep.1.00662. 10.1530/rep.1.00662 16123234

